# [Retracted] Hypoxia‑induced overexpression of DEC1 is regulated by HIF‑1α in hepatocellular carcinoma

**DOI:** 10.3892/or.2024.8850

**Published:** 2024-12-02

**Authors:** Wanshan Ma, Xiaohong Shi, Sumei Lu, Linlin Wu, Yunshan Wang

Oncol Rep 30: 2957–2962, 2013; DOI: 10.3892/or.2013.2774

Further to the publication of the above paper, a concerned reader drew to our attention that, in Figs. 1A and 4A, the same loading controls for the RT-PCR experiments portrayed had apparently been incorporated into these figures, even though the experimental results (i.e., for the DEC1 bands) were otherwise different. In addition, the control GAPDH bands featured in the western blots in Figs. 3A and 3B for the SMMC-7721 and BRL-7402 cell lines respectively were strikingly similar, albeit the bands appeared to have been vertically flipped in Fig. 3A relative to Fig. 3B, and possibly stretched.

The Editorial Office subsequently investigated this matter, and were able to confirm the concerns of the reader. After having considered the various issues that have been brought to light with this paper, in spite of a request from the authors that a Corrigendum be published, the Editor of *Oncology Reports* has determined that the article should be retracted on account of a lack of overall confidence in the presented data. The authors were asked for an explanation to account for these concerns, but the Editorial Office did not receive a reply. The Editor apologizes to the readership for any inconvenience caused.

